# Progressive Supranuclear Palsy: Clinical Features and Neuroimaging in a Case Series

**DOI:** 10.7759/cureus.83588

**Published:** 2025-05-06

**Authors:** Favio M. Fimbres-Laborin, Roger Carrillo-Mezo, Ricardo Lopez de la Cruz, Marie-Catherine Boll

**Affiliations:** 1 Clinical Research Laboratory, Instituto Nacional de Neurología y Neurocirugía Manuel Velasco Suárez (MVS), Mexico City, MEX; 2 Neuroradiology, Instituto Nacional de Neurología y Neurocirugía Manuel Velasco Suárez (MVS), Mexico City, MEX

**Keywords:** clinical deficits scale, imaging biomarkers, mrpi, mrpi2.0, p/m, progressive supranuclear palsy, superior cerebellar peduncle

## Abstract

Progressive supranuclear palsy (PSP), a neurodegenerative disease with remarkable clinical diversity, lacks early and robust biomarkers to confirm the diagnosis in vivo. Given this, we analyzed magnetic resonance imaging studies in a series of patients admitted to a referral center after a comprehensive clinical examination. We had access to the complete clinical and radiologic data of 12 PSP patients. In all cases, initial symptoms included gait and speech abnormalities. A functional scale assessment was performed prior to visual assessment of neuroimaging studies and planimetric analysis. We found potentially useful neuroimaging-based indices derived from brainstem and ventricular measurements. When examining correlations between these indices and the most affected clinical domains of the disease, we observed that higher values in specific measurements involving the pons area and cerebellar peduncles were significantly correlated with more severe impairment in gait and speech in our case series.

## Introduction

Progressive supranuclear palsy (PSP) is a rare neurodegenerative disease. Clinically, it has been classified as atypical Parkinsonism and pathologically as a 4R tauopathy. The latter is characterized by progressive aggregation of misfolded tau protein that spreads to different areas of the brain [1,2]. PSP is a severe disease with heterogeneous manifestations, a progressive course, and still no specific treatment [3]. The classic clinical presentation, known as Richardson’s syndrome (PSP-RS), originally described by Steele, Richardson, and Olszewski in 1964 [4], is characterized by oculomotor dysfunction, especially vertical gaze palsy, postural instability with unprovoked falls, progressive axial and limb rigidity, and cognitive impairment, among other motor and non-motor symptoms [5]. In 2017, the diagnostic criteria for this disease were updated by the Movement Disorder Society (MDS), with different variants described in detail [6]. The classical form, PSP-RS, is by far the most common, followed by the Parkinsonism-associated form (PSP-P), the progressive gait freezing form (PSP-PGF), and a frontal dementia form (PSP-F). A clear definition of these phenotypes could be useful for early diagnosis and prognosis; however, definitive diagnosis of the disease still relies on postmortem studies [7].

Recently, the use of biomarkers in PSP has gained interest, particularly neuroimaging biomarkers, which have shown high sensitivity and specificity in diagnosing PSP and differentiating PSP from Parkinson’s disease [8]. Similarly, several planimetric biomarkers on MRI have demonstrated their usefulness in differentiating PSP from other atypical Parkinsonisms [9].

## Materials and methods

Clinical protocol

In this study, we evaluate a series of patients diagnosed with PSP in a referral center (the National Institute of Neurology and Neurosurgery of Mexico) for whom complete clinical and radiological information is available A cross-sectional, descriptive study was performed based on the records of patients diagnosed with PSP in the period 2017-2024. Complete clinical data, diagnostic criteria for probable PSP according to the latest criteria of the MDS [6], and an MRI study with conventional sequences and slice thickness of 3 mm were mandatory for inclusion. The protocol was approved by all relevant institutional review boards and started in the year of approval. Eligibility criteria included age at onset of 50 years or older and gradual progression of the cardinal symptoms of PSP (oculomotor dysfunction, postural instability, akinesia, and cognitive dysfunction). The other 12 core clinical features described in MDS were also examined. The first three symptoms reported in the medical history were recorded, and the severity of PSP was assessed using the Clinical Deficits Scale (CDS) to determine functional status in the year in which the neuroimaging studies were performed [10]. Patients in our series underwent all mandatory standardized MRI studies, and any other neurodegenerative disease was excluded. Radiological markers with higher diagnostic validation were sought, and morphometric measurements were performed in collaboration with an expert neuroradiologist.

Image acquisition and processing

**Figure 1:**
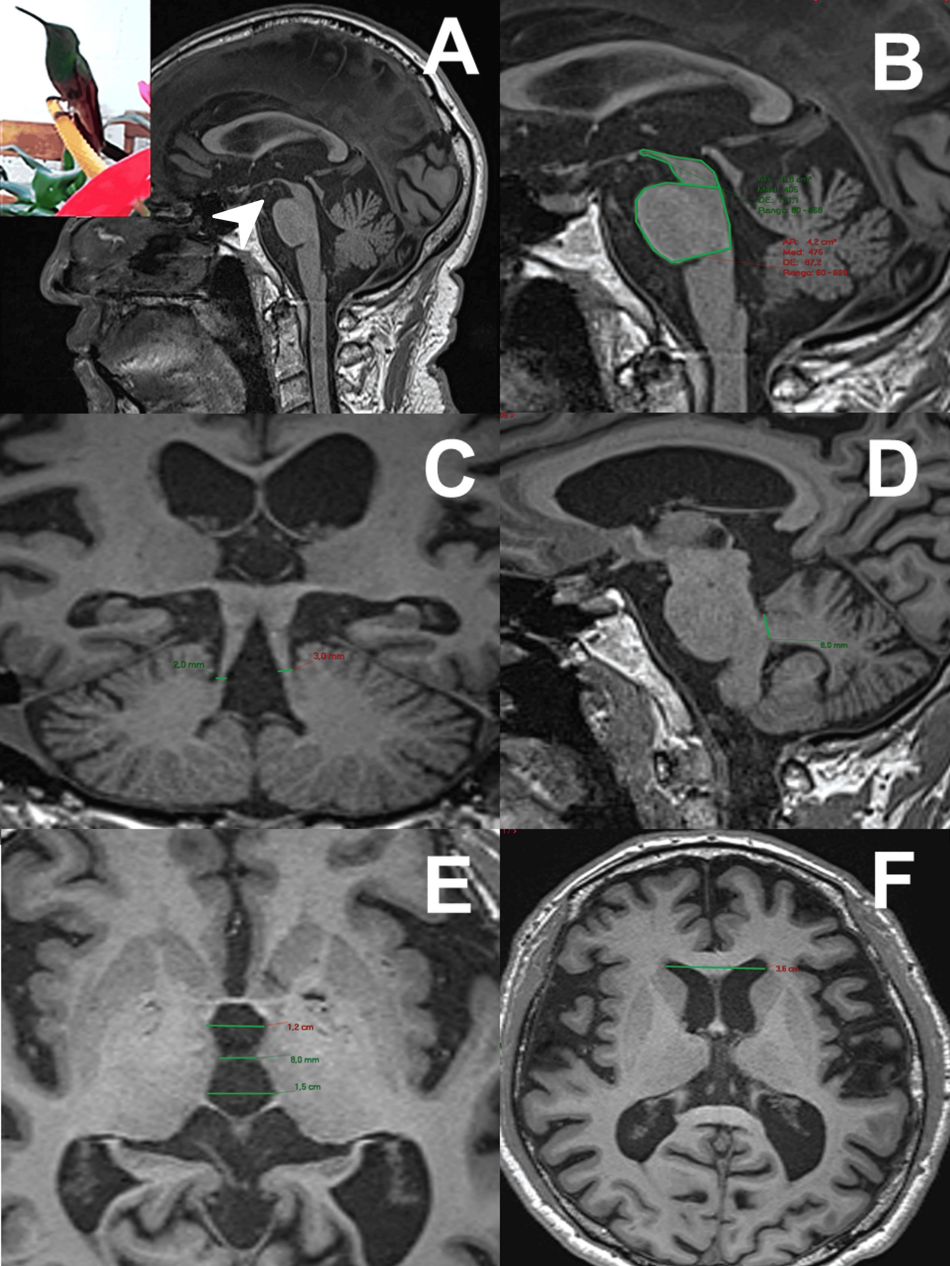
MRI visual assessment and planimetric measurements for MRPI and MRPI 2.0 calculation. A: hummingbird sign (arrow); B: sagittal midbrain area and sagittal pons area; C: coronal superior cerebellar peduncle width; D: sagittal medial cerebellar peduncle width; E: third ventricle; F: frontal horns of lateral ventricles. The hummingbird image used is derived from one of the patient cases included in our series, and the hummingbird photography was taken by Dr. Boll with a personal camera; both are original (not externally sourced). MRPI: magnetic resonance Parkinsonism index

Images were acquired in two magnetic resonance machines, a 3 T Siemens Skyra (Siemens Healthineers, Germany) and a 1.5 T Signa Explorer (GE Healthcare, Chicago, IL, USA). T1-based volumetric images were constructed using either the Siemens T1 MPRAGE technique in sagittal projection with reconstructions in the three planes or a General Electric T1 spoiled gradient echo (SPGR) technique with reconstructions in the three planes obtained from a conventional protocol (Figure 1B-1F).

These sequences were used to measure the area of the midbrain and pons in sagittal projection, the width of the middle cerebellar peduncle on both sides in sagittal projection, and the width of the inferior cerebellar peduncles in coronal projection. Three measurements of the third ventricle were taken in axial projection at the level of the intercommissural line. In the same plane, the bifrontal diameter was measured at the level of the foramen of Monro.

Several visual assessment signs have been proposed as adjuncts to MRI analysis, including the hummingbird sign, evaluated in a medial sagittal slice of the brainstem in the T1 sequence, which suggests midbrain atrophy, and the morning glory sign (Figure 1A) [11]. Four radiologic indices previously validated for the diagnosis of PSP were included in the planimetric analysis. These are the P/M index, the ratio of the midbrain sagittal area to the mid-sagittal area of the pons; the middle cerebellar peduncles/superior cerebellar peduncles (MCP/SCP) index, the ratio of the width of the superior cerebellar peduncle in the coronal plane (SCP) to the middle cerebellar peduncle in the sagittal plane (MCP); the magnetic resonance Parkinsonism index (MRPI index) [12], calculated as: \[\left( \frac{P}{M} \right) \times \left( \frac{\text{MCP}}{\text{SCP}} \right)\]The MRPI 2.0 index, the most recently proposed index, which includes the diameters of the third ventricle and frontal horns, calculated as: $\text{MRPI} \times \left( \frac{V3}{FH} \right)$ [13-15].

Statistical analysis

Our case series was obtained from a cohort of 39 patients. However, complete clinical and imaging information was available for less than half of these patients. A descriptive analysis of all variables was performed. Clinical-radiologic correlations were then examined. Data were collected in MS Excel (Microsoft Corporation, Redmond, Washington, United States) spreadsheets and analyzed using IBM SPSS Statistics for Windows, Version 27 (Released 2020; IBM Corp., Armonk, New York, United States).

## Results

Our case series included 12 patients, seven men (58.3%) and five women (41.6%), with a mean age of 60.25 ± 7.58 years. A diagnosis of probable PSP-RS was reported in nine patients (75%), probable PSP-P in two, and frontal variant (PSP-F) in one. The mean disease duration at the time of MRI was 2.33 ± 1.37 years. The first symptoms, in order of frequency, were gait disturbances (66%), including unprovoked falls (18.18%), bradykinesia (18.18%), postural instability (9.09%), gait freezing (3.03%), and other gait disturbances (18.18%), followed by speech problems (12.12%), cognitive impairment (9.09%), diplopia (6.06%), dysphagia (3.03%), and mood disorders (3.03%). Rigidity, vertical gaze palsy, and other movement disorders were reported later, with postural instability and falls being the most common.

The CDS functional score, measured in the same semester as the MRI studies, yielded a mean score of 12 ± 3.4 points (Table 1). We found no correlation between the time of progression and the functional scale. The most affected domains in the CDS were eye movements and gait. Cognitive impairment was documented in 58.3% of patients, and levodopa resistance in 83.3%. Of the 12 cases, 10 (83.3%) had hummingbird and/or morning glory signs on visual analysis.

**Table 1:** Clinical and functional characteristics of PSP patients. MDS: Movement Disorders Society; CDS: Clinical Deficit Scale; PSP-P: progressive supranuclear palsy-Parkinsonism; PSP-RS: progressive supranuclear palsy-Richardson’s syndrome; PSP-F: progressive supranuclear palsy-frontal

Subjects	Gender/Age at Onset (years)	Disease Duration	First Symptom	MDS-PSP2017 Diagnosis	CDS-Akinesia/Rigidity	CDS Bradyphrenia	CDS-Communication	CDS-Dysphagia	CDS-Eyes	CDS Fingers	CDS Gait	Total
1	M/47	4	Gait freezing	PSP-P	2	2	2	0	2	2	1	11
2	F/70	2	Unprovoked falls	PSP-P	1	3	1	0	3	2	2	12
3	F/58	1	Postural instability	PSP-RS	3	2	1	1	2	2	2	13
4	M/57	2	Diplopia	PSP-RS	3	2	1	2	1	2	1	12
5	M/53	4	Bradykinesia	PSP-RS	1	0	1	2	2	2	1	9
6	F/61	1	Bradykinesia	PSP-RS	1	1	1	0	2	1	1	7
7	M/56	1	Bradykinesia	PSP-RS	2	1	2	1	2	1	1	10
8	F/58	2	Weakness	PSP-RS	1	3	2	1	3	2	3	15
9	F/75	1	Unprovoked falls	PSP-RS	2	1	1	2	2	2	2	12
10	M/66	3	Mood disorder	PSP-RS	2	3	3	3	2	2	3	18
11	M/58	2	Speech difficulty	PSP-RS	0	0	2	1	2	1	2	8
12	M/64	5	Unprovoked falls	PSP-F	2	3	3	1	3	2	3	17

Results of the planimetric study

The mean P/M index was 4.94 ± 1.22 standard deviation (SD); the midbrain midsagittal area was 1.01 ± 0.32 SD, and the midsagittal pons area was 4.66 ± 0.40 SD. The mean MCP/SCP index was 3.80 ± 1.05, while the mean MRPI was 19.54 ± 8.83, and MRPI 2.0 was 4.42 ± 2.52; these latter results are highly suggestive of PSP according to Quattrone et al. [14]. Regarding the possible relationship between the imaging results and the functional score, we found no correlation between the total CDS values and the measurements. However, when the CDS was divided into its seven clinical domains, we found a clear tendency of relationship between MCP/SCP and CDS (Spearman’s coefficient = 0.567, P = 0.053) and a significant correlation with the gait domain (ρ = 0.664; P = 0.019) (Table 2). The communication and gait domains were also significantly correlated with the MRPI 2.0 (Spearman’s coefficient was 0.588 and 0.592, P = 0.044 and 0.042, respectively).

**Table 2:** Clinical-radiologic correlations (Spearman’s rho). CDS: Clinical Deficit Scale; P/M: mid-sagittal midbrain area/mid-sagittal pons area; MCP/SCP: middle cerebellar peduncles/superior cerebellar peduncles; MRPI: magnetic resonance Parkinsonism index; MRPI 2.0: magnetic resonance Parkinsonism index 2.0. * Correlation is significant at the 0.05 level (2-tailed).

CDS Domains	Statistical Analysis	P/M	MCP/SCP	MRPI	MRPI 2.0
CDS akinesia/rigidity	Correlation coefficient	-.046	-.138	-.130	-.222
	Sig. (2-tailed)	.886	.670	.688	.488
	N	12	12	12	12
CDS bradyphrenia	Correlation coefficient	.000	.068	-.058	-.022
	Sig. (2-tailed)	1	.833	.858	.947
	N	12	12	12	12
CDS communication	Correlation coefficient	.299	.567	.489	.588*
	Sig. (2-tailed)	.346	.054	.107	.044
	N	12	12	12	12
CDS dysphagia	Correlation coefficient	.406	.442	.361	.420
	Sig. (2-tailed)	.190	.150	.249	.174
	N	12	12	12	12
CDS eyes movements	Correlation coefficient	-.118	.158	-.004	.210
	Sig. (2-tailed)	.715	.623	.990	.512
	N	12	12	12	12
CDS finger dexterity	Correlation coefficient	-.168	-.170	-.307	-.251
	Sig. (2-tailed)	.602	.597	.332	.432
	N	12	12	12	12
CDS gait and balance	Correlation coefficient	.452	.664*	.507	.592*
	Sig. (2-tailed)	.140	.019	.093	.042
	N	12	12	12	12
CDS total	Correlation coefficient	.191	.344	.162	.232
	Sig. (2-tailed)	.552	.273	.615	.467
	N	12	12	12	12

## Discussion

PSP remains a diagnostic and therapeutic challenge due to its clinical heterogeneity and overlap with other neurodegenerative diseases. In this study, we describe the clinical and neuroimaging features in a case series with a diagnosis of probable PSP. It is worth mentioning that these patients underwent a fluorodeoxyglucose-positron emission tomography to analyze cerebral glucose metabolism, which helps to exclude Parkinson’s disease and other mimics. The results of our Mexican case series are consistent with those reported in European and American populations [15,16] and contribute to filling the information gap on clinical manifestations and neuroimaging biomarkers in a different geographical context. Our work highlights the importance of MRI in the diagnosis of PSP. Previous work has recommended planar morphometry for PSP diagnosis and found highly specific and sensitive markers such as P/M and MRPI (Figure 1B-1F) [17]. In our study, we confirmed the quality of these measurements and MRPI 2.0 since we found similar values, capable of discriminating PSP patients from healthy controls and Parkinson’s disease patients. The value of MRI as a diagnostic tool is irreplaceable [18], at least until the CSF biomarkers currently under investigation [19] and new specific radiotracers become available. The correlation between markers of focal atrophy and clinical elements such as gait is also described.

Most of the limitations of our study stem from its small sample size and non-analytic design. Regarding sample size, although cases were drawn from a cohort of 39 patients, complete clinical and imaging data were available for less than half of the original cohort, which may prevent us from studying rarer variants of the disease. Our study also lacks morphometric analysis in age- and sex-matched healthy controls and Parkinson’s disease patients. Finally, the lack of specific biomarkers and neuropathologic confirmation is a common obstacle in clinical studies due to the difficulty in accessing them.

Despite these limitations, our study lays the foundation for more extensive and collaborative research in the field of neurodegenerative disorders in Latin America. The establishment of national or regional registries to characterize the epidemiology of PSP and other tauopathies in different ethnic groups should be a priority. In addition, longitudinal studies involving the systematic collection of clinical, genetic, and imaging data, as well as efforts to conduct postmortem studies, would be valuable. These initiatives could contribute not only to early diagnosis but also to the development of disease-modifying therapies.

## Conclusions

In conclusion, this work highlights the importance of studying PSP in diverse settings and underscores the need to expand research to underrepresented populations. Although our series is small, our findings reinforce the value of specific imaging biomarkers as complementary tools in the differential diagnosis of PSP. Applying current clinical criteria and monitoring patients with clinimetrics and image review and analysis allowed us to correctly diagnose and find relevant clinical-radiologic correlations, especially between communication and gait abnormalities with MCP/SCP and MRPI 2.0. Emphasizing the quality of some morphometric markers may contribute to their inclusion in new diagnostic algorithms.
